# Connections between health research systems and decision-making spaces: lessons from the COVID-19 pandemic in the province of Québec, Canada

**DOI:** 10.1186/s12961-023-01053-y

**Published:** 2023-11-02

**Authors:** Pernelle Smits, Caroline Cambourieu, Mathieu Ouimet

**Affiliations:** 1 Departement de Management, Université Laval, Québec, Canada; 2 Université de Montréal, ESPUM, Montréal, Canada; 3 Departement de Sciences Politiques, Université Laval, Québec, Canada

**Keywords:** Ecosystem, Knowledge translation, Evidence, COVID-19, Decision-making

## Abstract

The coronavirus 2019 (COVID-19) pandemic allowed for exceptional decision-making power to be placed in the hands of public health departments. Data and information were widely disseminated in the media and on websites. While the improvement of pandemic management is still a learning curve, the ecosystem perspective – that is, the interconnection of academic health research systems and decision-making spaces – has received little attention. In this commentary, we look at the mechanisms in place, or not, in Canada for ensuring decision-making spheres can “speak” to academic research systems. We look at the thick walls that are still in place between health research systems and decision-making spaces. More precisely, we discuss three organizational flaws that we identified in the evidence-informed decision-making ecosystem of Québec and, more broadly, Canada. We introduce some inspiring measures that other countries have implemented to better link evidence and public health decision-making during health crises. The observed flaws and options are related to the vitality of early information sharing relays, the cross-sectional capacity to issue opinions, and the collection and integration of hard and soft data.

Ecosystems are organized systems of connected actors who work collectively. During the pandemic, the health emergency allowed for exceptional decision-making power, with the support of health data and information, to be placed in the hands of public health departments. One key to advancing a crisis management agenda is ensuring the efficiency of the evidence-informed decision-making ecosystem, that is, the combination of academic health research systems and decision-making spaces. This perspective on ecosystems as a means to optimize decision-making has received little attention from research funders, academic institutions, researchers and the public [1].

Much is known about strategies that foster connections between scientists and the decision-maker communities. Push, pull, link and exchange [2] strategies, and dedicated funds [3] are well-established actions that can promote knowledge transfer. Attention has also been given in recent years to individual and organizational capacities [4], to the diversity of data that should be given to decision-makers [5], both individuals and organizations, to strengthen evidence-informed decision-making [2, 6–8] and to indicators for evidence-informed policy-making [9]. That said, the goals and benefits of evidence-informed decision-making in public health still need to be improved [10].

In an emergency situation, the novelty of the event may lead to decisions being made on the basis of extrapolated data or historical information, as opposed to decisions being made on the basis of new knowledge. However, such data and information may not be particularly well suited to the emergency in question. Since the literature on the use of scientific data in political decision-making during health emergencies remained very poor before the coronavirus disease 2019 (COVID-19) pandemic [11], it is useful to look at experiences during the COVID-19 pandemic to identify issues related to the (non)existent links between health research systems and decision-making spaces.

Since COVID-19, several jurisdictions embraced the topic of bringing relevant knowledge to decision spaces. The WHO developed a framework with macroscopic criteria for routine use of evidence in such spaces [12]. The latest was tested on the management of the pandemic in several developed countries [13]. A review of ups and downs of the management of the pandemic in Canada highlighted fragmented responsibilities and calls for a shift of culture in data access [14].

Here, we examine issues related to the national ecosystem of evidence-informed decision-making. “Research evidence” is understood here as data and information that adopt a scientific methodology. It encompasses, among other things, data analytics, evaluations, behavioural/implementation research, qualitative research, synthesis and technology assessment/cost effectiveness. “Decisions” refer to decisions made by government policy-makers. “Evidence/decision ecosystem” refers to the ecosystem of research evidence/government policy-making.

To the best of our knowledge, systems thinking at the level of an evidence/decision ecosystem is just emerging [15, 16]. Two structures [17, 18] and one country, South Africa, were examined in studies conducted pre-pandemic [19]. The COVID-19 pandemic brought a resurgence in interest on closely related topics, including two panel discussions on evidence support ecosystems at the WHO Global Evidence-to-Policy (E2P) Summit in 2021 [20], a framework on sustainable use of evidence to inform decision-making [12], recommendations on national evidence infrastructure by the Global Commission on Evidence to Address Societal Challenges in Canada [21] and a call for projects on research governance by the Réseau Québécois COVID – Pandémie (RQCP) [22].

Our examination of evidence ecosystems focuses on the steps that should be followed to produce, translate and then linearly use evidence. However, decision-making, at its core, is also political and organic. Deficient links in the ecosystem may hamper its effectiveness and lead to harmful consequences, such as mortality, morbidity, negative impacts on social inequalities and additional costs.

Developments related to the evidence/decision ecosystem take evidence as a core concept, hence their name “evidence ecosystem”. Recommendations to build stronger evidence ecosystems cover the relevancy and quality of evidence, clarifying the information needs of decision-makers, and developing units and resources to support evidence and implement evidence into decision-making. In our view, this conception is still too linear, despite the openness to feedback from decision-makers. Much smaller consideration, if any, is given to institutionalized mechanisms that not only push research systems (evidence, researchers and so on) into decision-making spaces (decision-makers, decision cycles, existing workflows and so on), but push decision-making spaces into research systems. The processual approach at the nexus is what is of interest here – the in-between evidence and decision, so to speak.

This commentary takes a managerial and organizational view of the pandemic. We argue that beyond COVID-19 initiatives such as the production of policy briefs, the sustainability of such collective efforts to bring evidence into decision-making in times of crisis relies upon strong processes located at the nexus between decision-making spaces and (health) research systems. This paper looks at the thick walls that are still in place between health research systems and decision-making spaces. More precisely, we discuss three organizational flaws that we identified in the evidence-informed decision-making ecosystem in Québec and, more broadly, in Canada. We introduce some inspiring measures that other countries have implemented to better link evidence and public health decision-making during health crises. The observed flaws and options are related to the vitality of early information sharing relays, the cross-sectional capacity to issue opinions, and the collection and integration of hard and soft data.

In short, this commentary focuses on the structure – others would say institutionalization [23] – of the nexus that connects two environments, health research systems and decision-making spaces.

## Ensuring the vitality of early information sharing relays in the ecosystem

Jurisdictions responsible for public health are expected to anticipate crises. Starting in the late 2000s, international experts warned that various signals indicated that the emergence of new pandemic strains was very likely, making it possible to grasp the possibility that a pandemic could occur [24, 25]. To deal with the unpredictable nature of pandemics, decision-makers usually have at their disposal up-to-date and tested response plans, pandemic simulation exercises, and monitoring and risk-anticipation tools to pick up signals. The COVID-19 pandemic revealed significant gaps in such health monitoring. In short, surveillance systems exist in Canada and Québec but have been under performing during the pandemic.

### Canada

The Global Public Health Information Network (GPHIN) was created by the Public Health Agency of Canada in collaboration with the WHO in the late 1990s to be a primary source for the detection and early warning of contagious diseases for agencies in several countries. However, in 2019, the GPHIN failed to detect clues about the emergence of COVID-19 [26]. Overall, the risk was considered low until March 2020. A posteriori, it is noted that the detection capacity of GPHIN weakened starting in 2010; the government questioned its role in 2014, it focused on internal risks within Canada and the work of scientists lost its influence [26, 27]. Furthermore, although the GPHIN is a world-class network, some Québec health ministers knew very little or nothing at all about the network (personal communication). It was not until June 2021 that the Public Health Agency of Canada revived it [28].

### Québec

At the provincial level, there has been a plan in place to fight an influenza pandemic since 2006: the Québec Pandemic Influenza Plan – Health Mission [29]. However, it has never been updated and few of its strategies have been implemented, including the monitoring and updating of virological and epidemiological data based on the evolution of knowledge. It would take until February 2020 to release the first newsletter on the COVID-19 pandemic. Another strategy mentioned in this plan is the creation of an automated system that would allow for the consultation of real-time data and the integrated analysis of all data.

In short, pandemic response plans in Canada and Québec had not ever been rigorously evaluated or updated before the start of the COVID-19 pandemic [26, 27, 29, 30], and were even unknown to decision-makers [27].

Globally, various mechanisms for the early detection of epidemics make it possible to anticipate health crises. Early detection tools for health events target infectious diseases that pose a high level of risk to humans before these health emergencies become epidemics, including the Epidemic Intelligence from Open Sources, headed by the WHO and the European Commission (EC). Real-time visualization of emerging infectious threats to public health is facilitated by tools such as HealthMap in the United States and the EC’s Medical Information System (MediSys). Many tools translate evidence into a format suitable for decision-makers: Eurosurveillance provides the European public health community with an open-access platform for exchanging relevant monitoring results, the Knowledge for Policy (K4P) interface is operated by the EC, there are knowledge services and knowledge brokers for policy-makers, and the What Works Network brings together nine specialized research centres in the United Kingdom, and more.

Despite all these tools in different parts of the world, early action supported by detection tools still needs to be strengthened. The tools may be completely or little known to some decision-makers, and they are unnecessary and expensive if not used. There seems to be a lack of vitality in the devices used for capturing events and information needed by decision-makers. Thus, in the United States, the first person to be notified of the start of the crisis, in December 2019, was Dr. Marjorie Pollack, deputy editor of ProMed, a website run by the International Society for Infectious Diseases that advises healthcare professionals globally on emerging diseases. She was notified by one of her contacts, who lived in Taiwan and was active on Chinese social networks (Weibo). Dr. Pollack informed network members on 30 December 2019 [31]. Dr. Pollack can be seen as a transmission belt, a relay between the available evidence and the actors making decisions [27].

On the one hand, there are many systems for monitoring and producing support material for decision-makers. On the other hand, there is a need to ensure health security. These two areas seem to have connected only with difficulty and in slow motion at the start of the pandemic. This highlights a key missing element: the vitality of the relays between decision-making spaces and evidence systems during times of crisis.

The importance of the vitality of the evidence/policy ecosystem is presented in relation to the problem identification stage of the policy cycle. It would be possible to extend this reflection on ecosystem vitality to each relevant stage of the policy cycle with questions such as “Once a problem was identified, what mechanisms ensured the efficiency of best-available systematic reviews about pandemic management across decision-making bodies?”, “Once reliable evidence emerged as being in opposition to earlier decision-making, what mechanisms ensured the adjustment of decisions?” or “If a decision rippled into adverse health consequences, what mechanisms enabled researchers to pivot to emerging issues?”, and so on.

## Integrating soft and hard data in the ecosystem

The existence of event-based surveillance systems has not kept decision-makers from being confronted with a lack of clear, unambiguous and readily available evidence to characterize risks and make effective decisions [10] when needed. In times of crisis, decision-makers take two imperatives into account: the timeliness of action needed and the unpredictability of the situation. Decision-making takes place in dynamic and uncertain contexts (the behaviour of the virus, its infectivity, its dangerousness, the immune response generated and so on). Decision-makers cannot wait to have all the information needed before acting, as this might exacerbate the crisis and raise the risk of loss of control, negatively affecting the population. It seems unavoidable that the national ecosystem must consider both soft data, that is, data contextualized locally or stemming from social sciences, and hard data, that is, data stemming from international sources or the hard sciences.

Indeed, public health policy-makers usually resort to a wide range of contextualized local data and information sources [8, 32], such as epidemiological data, citizen queries, networks of “experts” (friends, colleagues, other politicians, consultants) [33], previous experience [10, 11] and even intuition and reasoning such as “it has worked before” or “they do this in other places” [8]. In addition, decision-makers consider that data becomes evidence for decision-making when contextualized, which is a step beyond using data generated using a scientific methodology [11]. In times of crisis, these types of contextualized data combine more or less harmoniously with noncontextualized data from recognized sources of authority.

### Québec

In autumn 2020, Québec’s public health body (the Institut national de santé publique du Québec, or INSPQ) and the WHO recommended half-day school attendance for fourth- and fifth-year high school students in areas where the cumulative number of confirmed COVID-19 cases was very high. The Québec government rejected this recommendation because it did not consider data specific to the context of intervention, namely, the pre-existing shortage of teachers and classrooms. Later, another contextual solution was designed based on administrative data from sources, including the educational and employment sectors. The chosen approach was alternate-day school attendance, with students being in class one day and having distance education from home the next day. Many agree that, beyond interventions based on universal public health approaches and international data, there is a need to make decisions rooted in the context and heterogeneity of living environments [34].

In times of crisis, data from specific health disciplines combine more or less harmoniously with data from the social sciences. It would have been helpful to draw on and integrate findings from a variety of fields when analysing the pandemic’s impact on seniors’ residences and how to manage it, including epidemiological data for people over age 65 years, organizational science for creating mobile teams, gerontology for optimizing modalities of care and social work for adapting living environments in residential centres. Regarding the pandemic in Québec, there was a wide effort to bring best evidence to front line workers [18] but governmental policy-makers did not seem to be fed with the same intensity with evidence about the management and organizational measures to protect older populations at greater risk. Instead, a team of experts was mobilized from medical disciplines [35]. Presumably, data on the pandemic management for the elderly would have been helpful to policy-makers.

### Canada

At the federal level, information is fed ministry by ministry, but no opinion forum provided an overview, so information remained fragmented before the COVID-19 pandemic hit. There have been several initiatives in Canada aimed at pulling together best-available evidence, including the COVID-19 Evidence Network to Support Decision-making (COVID-END), reviews of best-available evidence and scans of emerging issues, policy briefings from the Royal Academy of Sciences and the Canadian Network of COVID-19 Clinical Trials Networks. Of particular interest is knowing how to translate the efficacy of these platforms into an evidence/policy ecosystem that is sustainable, that is, capable of managing the next pandemic.

Some authors suggest reassessing those situations that, during health crises, favour the selection of evidence from biomedical research over other disciplines, such as social and political sciences [10]. Indeed, since epidemics of infectious diseases are due to multiple underlying factors, it justifies considering the potential contributions of data from the social, economic, political, biological and environmental fields [10]. It would be helpful to select and integrate very disparate data sources and then send to decision-makers those that are crucial and strategic, depending on, for example, the type of transmission, what populations and regions are most vulnerable and the appearance of vaccine-resistant variants. It is also important to integrate multiple disciplines, such as molecular biology for the sequencing of variants, gerontology for outbreak management in establishments for the elderly and animal health for zoonoses in breeding farms. A good example is the Master Question List for COVID-19, published biweekly by the Science and Technology Division of the United States Department of Homeland Security [36]. The list includes answers on clinical issues related to the disease and its detection, and other varied topics such as status of the disease in domestic animals, the effectiveness of public health measures, predictive models and more. It aims to provide decision-makers with reliable references on COVID-19 in a quick and operational format and to answer questions raised by decision-makers themselves.

Creating a dialogue between evidence and decision-making relies at least in part on mechanisms such as summary lists or teams of hard- and soft-evidence experts, the general principle being to the exposure to heterogeneity in disciplinary profiles.

## Improving the ecosystem’s permeability through resources with dual skills

Highly recognized structures promote the linkage between evidence and decision-making. One example of this is the guiding principle "evidence comes first" in the Action Plan to Strengthen the Use of Evidence, Information and Research for Policy-Making in the WHO European Region [37]. However, applying this principle involves major challenges for decision-makers who are under pressure and researchers who do not have all the answers. For scientists, there is no clear path to follow when engaging in policy-making calling for multiple disciplinary fields [38]. For decision-makers who are used to thinking in terms of consensus, there is great complexity in obtaining scientific results in a context of changing data [10]. It makes the exercise of linking decisions to scientific data uncomfortable. However, there is one person who dedicates themself solely to this task, that works at the intersection of two types of logic – the truth of the majority or the most vocal, and the truth of the facts – and that is the knowledge broker. However, this bridge builder typically works in a stable context, not a time of crisis; and crisis management requires additional skills.

### Canada

At the federal level, the institutionalization of this permeability can take the form of mechanisms such as ministerial scientific advisers. In 2019, Canada’s Chief Science Advisor established ministerial science advisors, who work closely with senior officials in federal departments but do not participate in their day-to-day activities. They provide objective feedback to senior managers and decision-makers after evaluating various sources of information, and facilitate the integration of evidence into decision-making processes [39]. These advisors can be supplemented by scientific experts who adopt a broader perspective and bring in social, economic, cultural and family dimensions [10]. The federal government’s current structure has yet to be implemented fully.

### Québec

Resources dedicated to the ecosystem of evidence-informed decisions are nonexistent or invisible in Québec. Starting in the late 1990s, a broad movement towards outsourcing ministerial research functions significantly weakened the research and evaluation capacities of the Ministry of Health and Social Services (MSSS). Since the state of research departments in Québec’s research institutes and centres and in the MSSS have not been assessed since then, it is difficult to know the nature of their practices.

However, a number of initiatives have had a favourable influence on the links between evidence and decision-making in Québec, including the creation of the advisory position of Chief Scientist of Québec in 2011, a concerted action programme between the ministries and research, and the arrival of advisory bodies such as the National Institute for Excellence in Health and Social Services (created in 2011) and the INSPQ (created in 1998).

Caution must be exercised with respect to the proximity of such agencies to the executive branch and possible biased forecasts about government preferences [40]. Moreover, at this time, there are no ministerial scientific advisers like there are at the federal level in Canada, or in the United Kingdom or New Zealand [41]. There no longer seems to be any symbolic role, nor capacity to bridge decision-making and evidence, within ministerial structures at the provincial level.

Such capabilities, when created in organizations, can fulfill several tasks. This is the case for chief data officers in the public and private sector, whose role is to support the reaching of objectives through evidence [42]. They combine data expertise and experience in managing organizations. The presence of resources with these dual capacities in the government apparatus, or the presence of resources with a close and independent connection with the government apparatus, would make it possible to understand the scope of political demands and scientific advances, promote calls for studies and define the questions and realities to be elucidated.

## Conclusions

The relationship between research evidence systems and decision-making spaces in the context of a health crisis has been little studied. It deserves greater attention, notably to ensure that scientific data and information in these contexts allows for rapid and effective crisis management [10]. To date, the managerial and organizational realities underlying the interface between evidence systems and decision-making spaces remain under explored, although some very recent publications do refer to national and global evidence infrastructures [20].

Note that decision-makers include government policy-makers, organizational leaders and citizens and professionals, and since best-available evidence based on scientific method is numerous [21], it can be assumed that each will favour certain types of evidence over others.

Our first observation is that the existence of anticipatory tools, such as risk management plans or event detection systems, does not guarantee their timely mobilization. Knowledge of these anticipatory tools, an understanding of them and using them appropriately require unique know-how and expertise by individuals wanting to harness these tools. In the absence of mechanisms and actors to ensure their vitality, evidence might risk being useless, while also creating a false sense of security. We therefore note that the emphasis must be on institutionalizing the vitality of the evidence/decision ecosystem at relevant stages in the policy cycle.

Our second observation is that the diversity of research evidence available to government policy-makers in times of pandemic adds to the high level of uncertainty about the situation and its evolution. Thus, resources and mechanisms should make it possible to integrate research evidence from various disciplines, hard and soft, to give them meaning for decision-makers and answers to the variety of situations they face. One of the underlying issues is the selection of sources and methods for data and information integration. Care must be taken that the government does not make these choices based on political and strategic parameters, that contradictory data and information is countered by subsidizing the media and their fact checkers, and that sources that share the decision-maker’s own predispositions are not overly influential ([43, 44], p. 205).

We wish to stress the universality of knowledge, that is, the ontology of knowledge, that goes beyond disciplinary lines, as well as to the habits of decision-making and the attention paid to non-mainstream research evidence. As decision-makers include government policy-makers, organizational leaders, citizens and professionals, and since best-available evidence based on scientific method is numerous [21], it can be assumed that each will favour certain types of evidence over others. Furthermore, there will be biases in the evidence/decision ecosystem in relation to certain types of evidence or certain types of decision-makers.

Our final observation is that, during a health crisis, the health system’s capacity to react in real-time is under pressure. Delays are caused by the unknown, the virus’s behaviour, infectivity, dangerousness, immune response and so on. Other delays seem to stem more from the internal capacity to organize and reorganize quickly, which is difficult when many levels are involved in the response (the federal government in charge of managing international arrivals versus the regional health bodies overseeing seniors residences). One path to consider is centralizing human resources with cross-sectoral skills to handle both the political and scientific points of view, for example by appointing an independent advisor to parliament. The success of the evidence/decision ecosystem lies in the independence of such structures. Research evidence might be produced without conflicts of interest and decisions might be taken from government policy-makers without any link to particular lobbies, but if the nexus that connects them both is not free from conflicts of interest, the entire ecosystem will lack independence.

Our objective in this commentary is not to provide a complete picture of each institution, mechanism and tool involved in the linking of the government policy-making and research evidence arenas. From a pragmatic point of view, at the time of writing (mid-2022), the health and social services network staff is still managing the pandemic and does not have time to answer uncomfortable questions in any detailed way. No one from the provincial or federal ministry of health would speak at length about the effectiveness of the internal mechanisms put in place and mobilized during the pandemic. For some, this was because they were not allowed to spend time on any form of research study.

Finally, given that we do not present any ecosystems as such, our inventory cannot be exhaustive. It was not a question of validating whether the situation in Québec or Canada corresponds to some pre-established framework that should be followed on a normative basis. This article complements the lessons learned from pandemic management in studies that examine the issues of care management and service delivery operations, along with their management and governance [1, 45].

This article makes several contributions to COVID-19 pandemic management and understanding evidence/decision ecosystems. It also opens up several avenues for reflection. This commentary brings a complementary perspective to the macroscopic-level WHO checklist for evidence-informed policy-making. We comment on dynamics that apply to middle-level management. Hanney et al. [13] present the benefits of coordination and integration within national health research systems, especially regarding vaccine and drug development. Our commentary adds some intangibles to improve the long-term management of those systems that facilitate coordination and integration of policy spaces and academic research systems.

Several elements are known to play a role in using research evidence in decision-making processes; a primary one is the lack of clear, unambiguous and rapidly available data and information on the characterization of risks and the effectiveness of intervention measures [8, 10]. Our article points out that, since the nature of data in times of crisis is unclear, hard and soft data from several disciplines are needed to combine research evidence, contextualize this evidence and promote rapid decision-making. We argue that hard and soft data must be integrated into the deployed mechanisms, even if the evidence do not align at first sight. The conditions conducive to joint work between several disciplines and with non-mainstream research evidence in times of pandemic should be explored.

It is widely accepted that risk prevention and anticipation are essential phases in managing a pandemic [8, 10]. We add that the tools and plans theoretically supposed to facilitate communication between actors remain of interest only if the actors bring them to life. Maintaining the vitality of communication tools, therefore, remains central. The appropriate profile of individuals most suited to bring vitality to such anticipation tools is a topic for further research.

The Knowledge Management for Policy (KPM) initiative, introduced by the European Commission’s Joint Research Centre, identifies eight attributes to maximize the value and impact of research when shaping evidence-based policies: synthesize research, manage communities of experts, understand policy and science, ensure effective people-to-people skills, engage with citizens and stakeholders, communicate scientific knowledge, monitor and evaluate, and advise policy-makers [46]. These attributes can be applied to each stage of the policy cycle. We add that the relays between decision-making and research evidence spaces should help strengthen such ecosystems. Such relays will help break down, or at least make more porous, the walls between research and decision-making. They could also be a source of problems due to, for example, researchers wearing many hats and having conflicts of interest, or researchers self-closeting for fear of retribution because their findings diverge from government thinking on problem definition and solutions. The independence of research in the context of a crisis should be explored [47].

Finally, the interest in managing pandemics based on research evidence is part of a tradition dating back several decades in which the objective is to identify the best methods for transferring evidence to the field of decision-making, as underscored in 2021 in the WHO report, Rapid Response: Knowledge Translation Mechanisms to Translate Evidence into Public Health Policy in Emergencies. Our article draws on another perspective: not that of seeing two entities separately, but that of thinking of a third link, where both entities influence each other in a bi-directional manner. We started to explore the connections that unite (or not) these two spaces, and we suggest further studies on ecosystem governance, that is, the bringing together evidence production space and the decision-making space.

Further research on organizational and managerial good practices and flaws in the evidence/policy ecosystem are needed. For example: Which mechanisms allow for a sustainable and independent nexus between evidence and policy? Which mechanisms are independent from the rotation of human resources in the production of evidence and in the uptake by decision-makers? Do the mechanisms currently in place cover key junctures in the policy cycle and agenda?

## Data Availability

Not applicable.
